# Dual checkpoint inhibitor-associated eosinophilic enteritis

**DOI:** 10.1186/s40425-019-0743-5

**Published:** 2019-11-15

**Authors:** J. Yang, S. M. Lagana, Y. M. Saenger, R. D. Carvajal

**Affiliations:** 1 Department of Medicine, Memorial Sloan Kettering Cancer Center, New York, NY USA; 2 Department of Pathology and Cell Biology, Columbia University Irving Medical Center, New York, NY USA; 3 Herbert Irving Comprehensive Cancer Center, Columbia University Irving Medical Center, New York, NY USA

**Keywords:** Melanoma, Checkpoint inhibition, Eosinophilia, Eosinophilic enteritis

## Abstract

**Background:**

Eosinophilia has been reported as a rare, new biological effect of immune checkpoint inhibition that may be associated with improved treatment response and the development of immune-related adverse events.

**Case presentation:**

We report a case of dual checkpoint inhibitor-associated hypereosinophilia and eosinophilic enteritis in a patient with advanced cutaneous melanoma. Rapid resolution of peripheral eosinophilia and associated symptoms was achieved with steroids alone.

**Conclusions:**

Immune checkpoint inhibition can trigger inflammation in virtually any organ in the body, leading to diverse clinical manifestations. To our knowledge, this is the first case report of eosinophilic enteritis due to ipilimumab plus nivolumab.

## Introduction

Immune checkpoint inhibition with anti-PD-1 (programmed death 1) and anti-CLTA-4 (cytotoxic T-lymphocyte associated protein 4) agents has revolutionized the treatment of various cancers, but can be associated with a diverse range of immune-related adverse events including pneumonitis, colitis, and rare cases of myocarditis. Eosinophilic enteritis is a rare primary eosinophilic gastrointestinal disorder, first described in 1937, characterized by gastrointestinal symptoms in the presence of pathological eosinophilic infiltration of the intestinal wall without secondary causes of gut eosinophilia. To our knowledge, this is the first reported case of eosinophilic enteritis associated with ipilimumab plus nivolumab.

## Case presentation

A 68-year old Caucasian man was referred to our medical oncology clinic in August 2017 for management of a stage IIIB (AJCC version 7) cutaneous melanoma. He was diagnosed with a 2 mm thick melanoma over the right scalp in December 2012 that was excised. Sentinel lymph node biopsy was negative. He remained disease-free until August 2017 when he noticed a pruritic scalp nodule located approximately 2 cm from the prior skin graft that was biopsy-proven to be an in-transit recurrence of the melanoma. He underwent repeat excision, with pathological staging demonstrating a stage IIIB (pT4apN2cM0) melanoma. Molecular testing was notable for a *GNA11* Q209L mutation that was also found in the previous specimen from 2012. MRI brain and CT chest, abdomen, and pelvis were negative for distant metastases. He was started on adjuvant pembrolizumab on 12/14/2017. Shortly after the first dose, he developed an enlarging right supraclavicular lymph node. Fine needle aspiration of the lymph node revealed metastatic melanoma. Repeat scans after 3 additional doses of pembrolizumab demonstrated new hepatic lesions. Treatment was intensified with the addition of talimogene laherparepvec (T-VEC) injections into the supraclavicular lymph node. He developed a maculopapular rash that was managed with topical hydrocortisone. There was significant decrease in the size of the injected lymph node with initially stable visceral disease, but MRI abdomen on 6/8/2018 after 5 concurrent doses of T-VEC and pembrolizumab showed interval growth in several hepatic lesions.

**Fig. 1:**
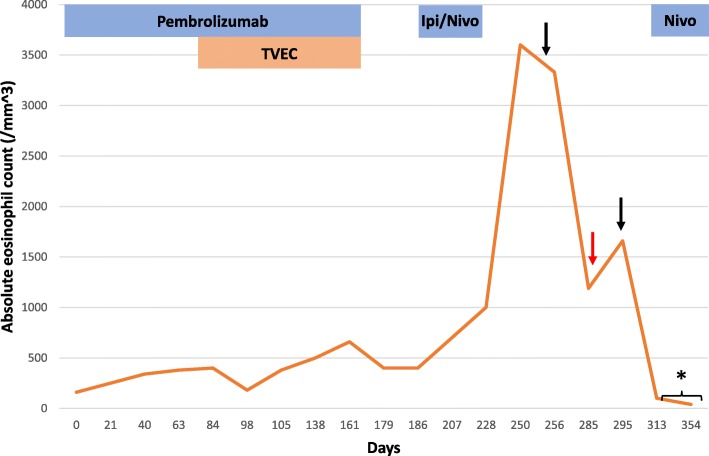
Absolute eosinophil count over treatment course. First black arrow – initiation of prednisone 1 mg/kg. Red arrow – steroid dose tapered down to prednisone 5 mg daily. Second black arrow – steroid dose increased to prednisone 15 mg daily. Asterisk – unknown eosinophil count

**Fig. 2:**
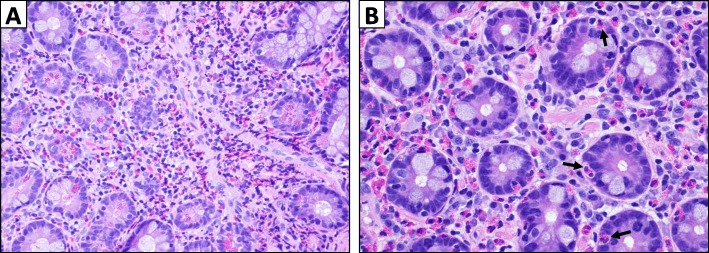
Duodenal biopsy showed extensive eosinophilic infiltrate. A – Greater than 100 eosinophils per high-power field, predominantly in lamina propria (hematoxylin and eosin, 40X view). B – Eosinophilic infiltration within duodenal crypts as indicated by black arrows (60X view)

He was transitioned to dual checkpoint inhibition with ipilimumab 3 mg/kg plus nivolumab 1 mg/kg and received his first dose on 6/21/2018. Blood work on 7/9/2018 was notable for an absolute eosinophil count (AEC) of 700/mm^3^ [normal range 30–350; peripheral eosinophilia defined as AEC > 500/mm^3^] (Fig. 1). He received 2 additional doses of ipilimumab plus nivolumab on 7/12/2018 and 8/1/2018 with concurrent stereotactic body radiation therapy (SBRT) to 2 liver lesions (50 Gy over 5 fractions to each lesion). He returned for his final dose of combination therapy on 8/23/18, but treatment was held for worsening pruritus, rash, non-productive cough, and new transaminitis (AST 60, ALT 151). Around this time, he also developed vague gastrointestinal symptoms consisting of abdominal pain, bloating, nausea, and diarrhea. His AEC continued to rise, peaking at 3600/mm^3^ on 8/21/2018. He did not have rectal bleeding or ascites. Stool and serologic studies were negative for parasitic infection. Restaging scans on 8/27/2018 showed further disease progression in the liver and the development of a new soft tissue paraspinal lesion. He was evaluated by a gastroenterologist and underwent an upper endoscopy which revealed no gross abnormalities, but biopsy of the duodenum revealed a prominent eosinophilic infiltrate (80–100 eosinophils per HPF) consistent with eosinophilic enteritis (Fig. 2).

He was started on prednisone 1 mg/kg daily with rapid improvement in his peripheral eosinophilia, rash, cough, and gastrointestinal symptoms. When the prednisone was tapered off, he developed recurrent symptoms with a concomitant rise in his AEC. He was restarted on prednisone 15 mg daily with normalization of his AEC. Subsequent scans demonstrated essentially stable disease for three months, although minor growth was noted in several cutaneous lesions. He received additional lines of therapy including off-label trametinib for *GNA11*-mutant melanoma, nivolumab monotherapy, and combination chemotherapy with carboplatin, vinblastine, and dacarbazine. The most recent AEC was normal off prednisone. Most recent imaging showed disease progression in the lung, and he was restarted on ipilimumab plus nivolumab in combination with T-VEC injections.

## Discussion

Primary eosinophilic gastrointestinal disorders encompass a group of rare diseases characterized by pathologic eosinophilic infiltration of the gastrointestinal tract in the absence of other identifiable causes of gut eosinophilia. Eosinophilic gastritis, enteritis, and gastroenteritis are generally grouped together due to clinical similarities, but it is unclear whether these represent distinct entities or share a common pathological process [1]. Abdominal pain, nausea, and vomiting are common presenting symptoms, and the majority of patients have peripheral eosinophilia. There are no consensus histopathologic criteria for diagnosis, but experts have proposed guidelines suggesting that greater than 25 eosinophils per high power field (HPF), in conjunction with eosinophilic acute cryptitis, is abnormal [2]. The diagnosis of eosinophilic enteritis additionally requires the exclusion of secondary causes of intestinal eosinophilia such as parasitic infections, inflammatory bowel disease, autoimmune vasculitides, and culprit drugs e.g., gold therapy, oral hypoglycemic agents, and non-steroidal anti-inflammatory drugs (NSAIDs) [3].

The molecular pathogenesis of eosinophilic gastroenteritis is thought to be mediated by a type 2 helper T cell (Th2)-driven immune response that triggers eosinophil chemotaxis and activation. Transcriptomic analysis of gastric biopsies obtained from patients with eosinophilic gastroenteritis reveals activation of the Th2 cytokine signaling pathways IL-4, IL-5, and IL-13. Upregulation of the chemokine CCL26 (eotaxin-3), a known eosinophil chemoattractant and downstream target of IL-4 and IL-13, further corroborates the central role of Th2-driven immunity [4–7]. Similar to our patient who has a history of multiple allergies to dust, pollen, latex, and several medications (penicillin, pregabalin, ibuprofen), affected individuals tend to have atopic phenotypes consisting of asthma, eczema, and allergies to food or medicine. In the case we present, checkpoint inhibition may have provoked a similar immune response in the duodenum leading to eosinophilic inflammation. Indeed, T cell co-stimulation through CD28 and B7–2 plays an important role in the Th2-mediated immune response that promotes bronchial asthma. Administration of a CTLA-4 immunoglobulin (fusion protein consisting of extracellular domain of CTLA-4 and a human γ-1 constant region) blocks this interaction and reduces eosinophil accumulation and Th2 cytokine production [8]. Thus, we may surmise that checkpoint inhibition with ipilimumab could trigger allergic conditions such as eosinophilic enteritis. Alternatively, the accumulation of gut eosinophils may have been secondary to a hypereosinophilic syndrome-like condition induced by immunotherapy.

Peripheral eosinophilia, long observed during the course of IL-2 therapy due to induction of IL-4 and IL-5 [9–11], was recently documented as a new, rare biological effect of checkpoint inhibition. A retrospective case series based on a French pharmacovigilance registry included 909 patients who received anti-PD-1 or anti-PD-L1 therapy between 2013 and 2016 [12]. A total of 26 patients (2.8%) were deemed to have immune-related eosinophilia, the majority of whom were treated for advanced melanoma. The median time to increase in eosinophil count was 3.0 months after the first cycle of therapy, with peak eosinophilia (median peak 1000/mm^3^) occurring after a median of 6.4 months. Notably, no patient developed any clinical manifestations related to eosinophilia. Our patient experienced a mild increase in AEC from 160/mm^3^ to > 300/mm^3^ approximately 1 month after initiating pembrolizumab monotherapy and had a second increase to 700/mm^3^ roughly 2 weeks after receiving the first dose of ipilimumab plus nivolumab. The peak AEC of 3600/mm^3^ occurred 2 months after dual checkpoint inhibitor treatment (Fig. 1). Two cases of drug reaction with eosinophilia and systemic symptoms (DRESS) syndrome due to immune checkpoint inhibition have also been reported [13, 14]. Although our patient did present with rash and systemic symptoms including cough and gastrointestinal complaints, DRESS syndrome seemed less likely in the absence of fever, lymphadenopathy, extensive rash covering the face and greater than 50% of the body surface area, and typical involvement of the kidney or liver. He had a mild transaminitis attributed to radiation therapy that resolved prior to initiation of steroids.

Prior reports additionally suggest that baseline elevation in eosinophil count [15, 16] or increase during treatment with checkpoint inhibition [17] may serve as predictive biomarkers of improved response and survival outcomes as well as increased risk of immune-related adverse events in melanoma patients. For example, in a retrospective analysis of 616 patients with advanced melanoma treated with pembrolizumab, baseline relative eosinophil count (REC) > 1.5% was an independent prognosticator of improved overall survival (median OS 19.6 months vs 5.8 months in patients with REC > 1.5 and < 1.5% respectively) [15]. Similarly, improved response rates and long-term disease control were observed in metastatic melanoma patients who experienced an increase in AEC of > 100/mm^3^ or had an AEC > 400/mm^3^ at 12 weeks after initiating anti-PD-1 therapy [17]. 22 of 73 patients (30%) with advanced melanoma treated with ipilimumab at a single institution developed hypereosinophilia (defined as an AEC > 400/mm^3^) during the course of their treatment [18]. An increase in AEC of greater than 100/mm^3^ between the first and second infusions was associated with longer survival (median OS 11.3 months versus 6.8 months, *p* = 0.012), and 73% of these patients had immune-related adverse events primarily involving the gastrointestinal tract. None of these adverse events were thought to be directly related to the hypereosinophilia, though biopsies of the affected organ were not obtained in all cases. Our patient had initial radiographic progression followed by several months of stable disease, but whether disease control was secondary to delayed immunotherapy response or receipt of other regimens such as trametinib and chemotherapy is unclear. Interestingly, a recent case report of hypereosinophilia in a non-small cell lung cancer patient who had lethal, hyperprogressive disease after a single dose of nivolumab highlights a more complex, context-specific role for eosinophils in which the immune modulatory effects of eosinophils may depend on which cytokines are present in the immediate surroundings [19]. TNF-α and IFN-у appear to enhance eosinophilic production of pro-inflammatory Th1-type chemokines such as CXCL9 and CXCL10 whereas TNF-α and IL-4 stimulate eosinophilic production of Th2-type chemokines, which sustain a more immunosuppressive tumor microenvironment.

The immunological role of eosinophils is not fully understood, but they are implicated in various immune processes including host immune response to helminthic infections, pathogenesis of atopic conditions, and tumor surveillance. Eosinophils may promote antitumor immunity by recruiting CD8+ T cells to the tumor microenvironment via secretion of chemoattractants (CCL5, CXCL9, and CXCL10) and normalization of tumor vasculature, thereby allowing for increased effector T cell infiltration [20]. Moreover, eosinophils may polarize tumor-associated macrophages toward an M1 phenotype [20] and mediate direct tumor cell lysis via the release of granule-associated cytotoxic proteins including major basic protein, eosinophil peroxidase, and eosinophil-derived neurotoxin. Indeed, IL-5 transgenic mice expressing high endogenous levels of eosinophils displayed resistance to the development of methylcholanthrene (MCA)-induced fibrosarcomas [21]. In the case we present, it remains unclear whether there was any correlation between the eosinophilia and period of stable disease given the patient received multiple subsequent lines of therapy.

## Conclusion

We present a case of dual checkpoint inhibitor-associated hypereosinophilia and eosinophilic enteritis in a patient with advanced cutaneous melanoma. Rapid resolution of peripheral eosinophilia and associated symptoms was achieved with steroids alone. To our knowledge, this is the first case report of a direct clinical manifestation of hypereosinophilia due to immunotherapy.

## Data Availability

N/A
